# A novel function prediction approach using protein overlap networks

**DOI:** 10.1186/1752-0509-7-61

**Published:** 2013-07-17

**Authors:** Shide Liang, Dandan Zheng, Daron M Standley, Huarong Guo, Chi Zhang

**Affiliations:** 1 Systems Immunology Lab, Immunology Frontier Research Center, Osaka University, Suita, Osaka 565-0871, Japan; 2 Department of Radiation Oncology, University of Nebraska Medical Center, Omaha, NE 68198, USA; 3 Department of Marine Biology, Ocean University of China, Qingdao 266003, P. R. China; 4 School of Biological Sciences, Center for Plant Science and Innovation, University of Nebraska, Lincoln, NE 68588, USA

**Keywords:** Protein overlap network, Protein function prediction, Composite network, Functional genomics

## Abstract

**Background:**

Construction of a reliable network remains the bottleneck for network-based protein function prediction. We built an artificial network model called protein overlap network (PON) for the entire genome of yeast, fly, worm, and human, respectively. Each node of the network represents a protein, and two proteins are connected if they share a domain according to InterPro database.

**Results:**

The function of a protein can be predicted by counting the occurrence frequency of GO (gene ontology) terms associated with domains of direct neighbors. The average success rate and coverage were 34.3% and 43.9%, respectively, for the test genomes, and were increased to 37.9% and 51.3% when a composite PON of the four species was used for the prediction. As a comparison, the success rate was 7.0% in the random control procedure. We also made predictions with GO term annotations of the second layer nodes using the composite network and obtained an impressive success rate (>30%) and coverage (>30%), even for small genomes. Further improvement was achieved by statistical analysis of manually annotated GO terms for each neighboring protein.

**Conclusions:**

The PONs are composed of dense modules accompanied by a few long distance connections. Based on the PONs, we developed multiple approaches effective for protein function prediction.

## Background

Proteins are basic functional components in any biological process. Discovery of the functions of an individual protein is therefore a critical step towards understanding biological processes and the complete biological system. Besides experimental studies, computational prediction plays an important role in current protein function investigation [1]. The general biochemical function of a protein can be inferred if the amino acid sequence or 3-D structure of the protein resembles another protein whose function is known [2-7]. The rationale for the homology-based method is that two proteins with a similar sequence or structure could evolve from a common ancestor and thus have similar functions. Although this homology-based method remains the most widely utilized computational tool for function assignment, some proteins identified from genome sequencing do not have any homologs that were functionally characterized in the previous studies. On the other hand, homologous proteins might also acquire different functions in evolution [8]. Thus computational methods other than the homology-based method are much in demand to improve the accuracy and coverage for protein function prediction.

Graph theoretical analysis can be used for predicting the function of uncharacterized proteins [9-11]. Briefly, genes or the products of genes, i.e. proteins, are represented as nodes in the network and two nodes are connected if they are in some type of association, e.g., protein-protein interaction (PPI). Possible functions of a protein can be assigned based on the frequently observed known functions of its immediate interacting partners [12]. Advanced approaches, such as defining the neighborhood of a protein with a radius of *n*[13], considering the shared neighborhood of a pair of proteins [14], transferring common annotations in a module to the uncharacterized members [15-19], were also developed over the years.

Unlike function inference methods, construction of a reliable network remains the bottleneck for network-based function prediction. High-throughput experimental methods such as two-hybrid techniques [20], affinity precipitation [21], and synthetic lethal screening [22,23] have been developed to construct PPI networks. Gene expression networks were built from gene pairs showing significant correlation of expression in microarray experiments [24,25]. In spite of wide applications, the high-throughput experimental methods often sacrifice specificity for scale. The constructed networks have a high level of false positives [26] and the predicted protein functions are even noisier.

The so-called genomic context (GC) methods, namely, gene fusion [27], gene order conservation [28], phylogenetic profiles [29], and operon rearrangement [30,31] exploit the genome sequences themselves to predict protein-protein associations. The derived information can be used for the construction of a PPI network. As a result, the network based approach showed better performance than the raw scores of GC-based methods in protein function prediction [10].

To a less extent, domain co-occurrence networks (DCN) were investigated for the prediction of protein and domain functions [32]. A protein domain is a continuous sequence within the protein that represents a structural, functional, and evolutional unit. A simple protein may contain a single domain, whereas 70% of eukaryotic proteins are composed of multiple domains [33]. In a DCN, two domains are connected by an edge if there is one protein containing both of them. Previous studies showed that DCNs are scale-free and small-world networks [34]. The domain function could be predicted by counting the occurrence frequency of GO terms associated with the neighboring nodes in a single-genome DCN while the function of a multiple-domain protein could be derived by integrating the predicted function of each domain. The prediction results were not improved by sophisticated algorithms such as χ^2^ method or support vector machine (SVM) learning [32].

As a counterpart to DCN, the protein overlap network (PON) provides another way for function prediction. In a PON, each node represents a protein and two nodes are connected with an edge if the proteins share a common domain. While a few efforts have been made to study the properties of DCNs [32,34-36], to our knowledge, there has been no detailed research report but a few brief comments about PONs [35]. In this study, we constructed the PONs with the entire genomes of individual organisms, studied the network properties, and made predictions of protein function with the composite PON of multiple genomes.

## Methods

### Data acquisition

The protein annotations were downloaded from InterPro [37], a database that integrates various resources, for the complete genomes of yeast (*S*. *cerevisiae*), fruit fly (*D*. *melanogaster*), worm (*C*. *elegans*), and human (*H*. *sapiens*) in April 2012. Every protein was annotated with domains retrieved from the Pfam database [38], and the functions of each domain were represented by their available GO (gene ontology) term annotations [39]. The four species were selected because they were all well-studied model organisms spanning comprehensive levels of complexity. We found 4,759 proteins possessing a UniProt ID [40] and Pfam annotation, and a total number of 2619 domains with 947 GO term annotations in yeast, as well as 12,933 proteins (3422 domains and 1114 GO terms) of fly, 15,433 proteins (3172 domains and 1093 GO terms) of worm, and 41,053 proteins (4290 domains and 1363 GO terms) of human. The GO term annotations were the same for the common domains contained in various proteins of the four species.

We also used UniProt-GOA (gene ontology annotation) database [41] for prediction and evaluation, in which GO terms were assigned to gene products using a combination of high-quality electronic mappings and manual curation. Even homologous proteins with the same domain composition might acquire various functions in evolution and thus annotated with different GO terms. The gene association files were downloaded from http://www.ebi.ac.uk/GOA/ for yeast, fly, worm, and human in April 2012.

### Network construction and property analysis

In our constructed PONs, each node represents one protein. Two nodes are connected by an edge if the corresponding proteins share a common domain defined by Pfam. The proteins and the domains acquired above were used to construct a PON for yeast, fly, worm, and human, respectively.

We investigated three main network properties for the PONs: degree and degree distribution, shortest path length, and clustering coefficient. The degree of a node is defined as the number of its immediate neighbors. The shortest path length between any two nodes is calculated with Dijsktra algorithm [42] using B-heaps scheme. The clustering coefficient of node *i* is defined as

(1) $$\mathrm{ci}=\frac{2\mathrm{ni}}{\mathrm{ki}\left(\mathrm{ki}-1\right)}$$

where *n*_*i*_ is the number of connected node pairs (edges) among nodes directly connected to the central node *n* and *k*_*i*_ is the number of neighboring nodes of the central node *n*.

### Protein function prediction with a domain-based method

We used the neighbor-counting method to retrieve GO terms associated with all neighbors of a query protein in the PON and ranked them by their occurrence frequencies. Instead of simply counting the number of protein nodes associated with a GO term, the occurrence frequency was calculated as the number of domains that were associated with the GO term and presented in the neighboring nodes. Naturally, GO terms associated with the common domains shared by a query protein and its neighbors should be considered more favorable than those associated with domains observed only in the neighbors. For the purpose of testing the prediction method, we assumed that the function was unknown for all domains in the query protein. Thus, unless specifically indicated, we made predictions using only domains that were not observed in the query protein, i.e., excluding the common domains. In cases where a GO term was associated with both the common domain and other domains in neighbors, the GO term was considered in prediction but the common domain was not counted in the occurrence frequency. The result was compared with the combination of annotated GO terms of all domains in the query protein. As a control to evaluate the prediction performance, we randomly switched GO terms belonging to two proteins and the corresponding domains throughout the whole genome.

### Protein function prediction with GOA database

Annotated GO terms obtained from the GOA database were statistically analyzed for neighboring nodes of the query protein. Those with a high occurrence frequency, i.e., associated with a large number of proteins, were selected and compared with the annotations of the query in the GOA database.

### Evaluation of protein function prediction

We used the success rate of prediction as the primary evaluation method. The success rate was defined as the ratio of correctly predicted GO terms to the total predictions. For the purpose of evaluation, we limited the number of predicted GO terms to be the same as that of the annotated GO terms in the query protein. Frequently, several GO terms had the same occurrence frequency around the cut-off and only a part of them were randomly selected to meet the criteria. We might also have a smaller number of GO terms associated with the neighboring proteins than the total annotations of the query protein and thus all of the analyzed GO terms constituted the prediction. In addition to the success rate, we also evaluated top 3 ranked GO terms, which means that the prediction was considered correct only if one of the three GO terms matched any annotation of the query protein. Similarly, the GO term with the highest occurrence frequency was evaluated for the top 1 GO term accuracy. A node was considered predictable if any of its neighboring nodes was annotated with one or more GO terms. Predictable nodes with observed GO term annotations were subject for evaluation and the coverage was calculated as the percentage of the evaluated nodes out of all nodes in the network.

## Results

### Statistical properties of protein overlap network

**Figure 1:**
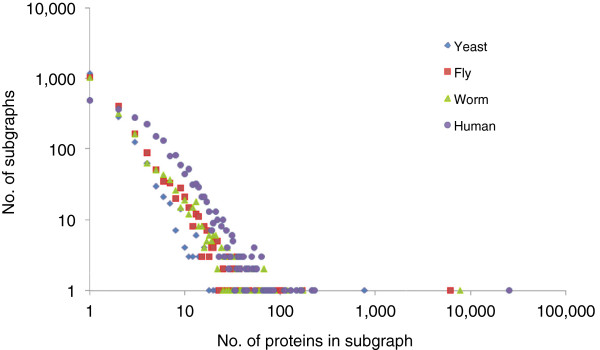
**Relationship between quantity and size of sub-graphs.** The PON of an entire genome was calculated.

We built PONs with proteins from the whole genomes of yeast, fly, worm, and human, respectively. The network usually contained many disconnected sub-graphs with one main sub-graph consisting of 50% of proteins except for the relatively small yeast PON, in which the main sub graph contained 16% of the total proteins and 24% of proteins were not connected to any other proteins (Figure 1).

**Figure 2:**
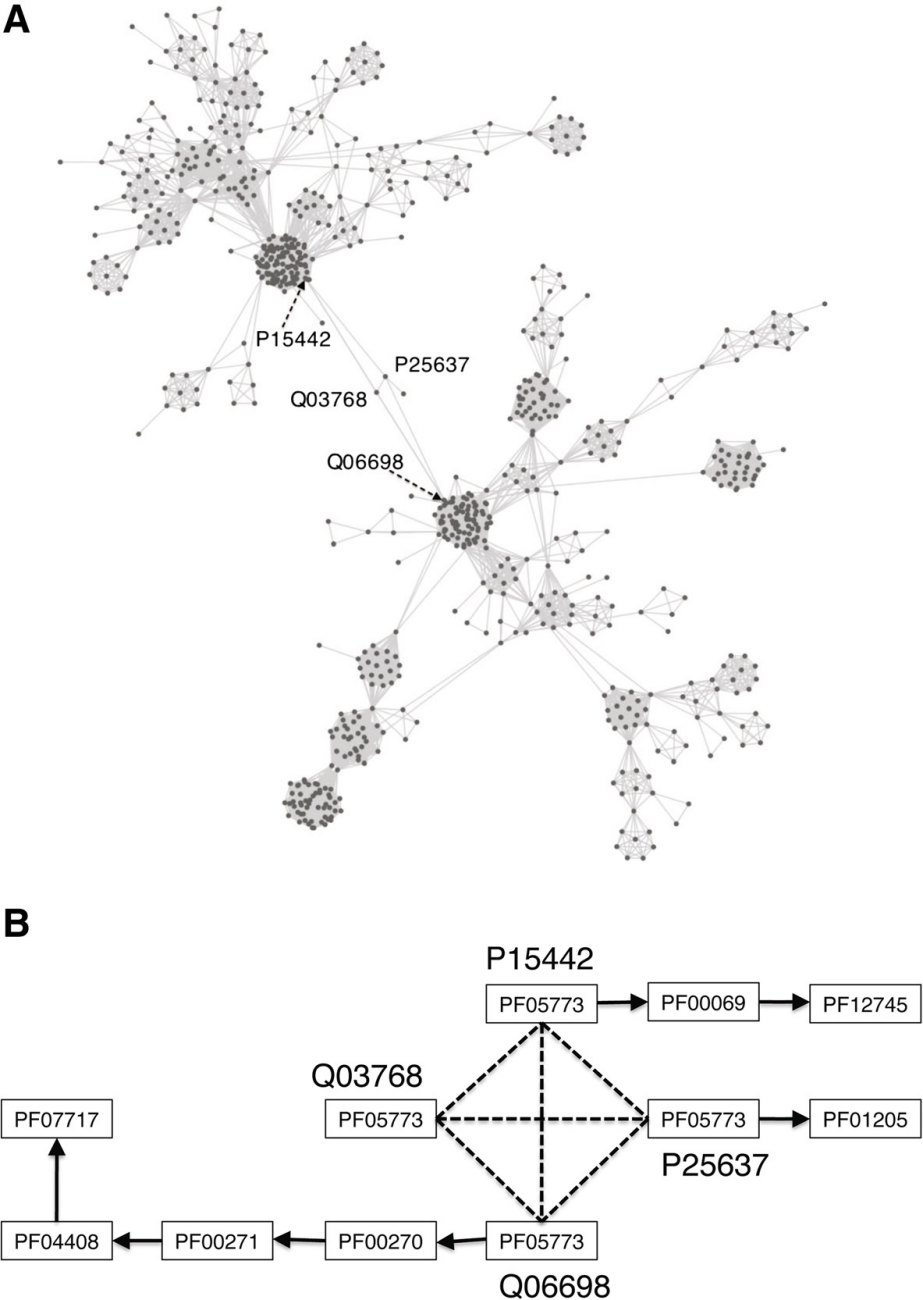
**Main sub-graph of yeast PON. (A)** Main sub-graph consisting of two modules. **(B)** Network connections between two modules. The dashed line represents the edge between two nodes and the arrow indicates the domain connection within a protein. Two modules in the graph are connected by domain RWD [Pfam: PF05773] associated with *protein binding function*. Pkinase [Pfam: PF0069, upper module], DEAD [Pfam: PF00270, lower module], and Helicase_C [Pfam: PF00271, lower module] are prevailing domains associated with *ATP binding function*. Only annotated domains in InterPro database were presented for proteins GCN2 [UniProt: P15442], GIR2 [UniProt: Q03768], IMPACT homolog [UniProt: P25637], and a putative ATP-dependent RNA helicase [UniProt: Q06698].

**Figure 3:**
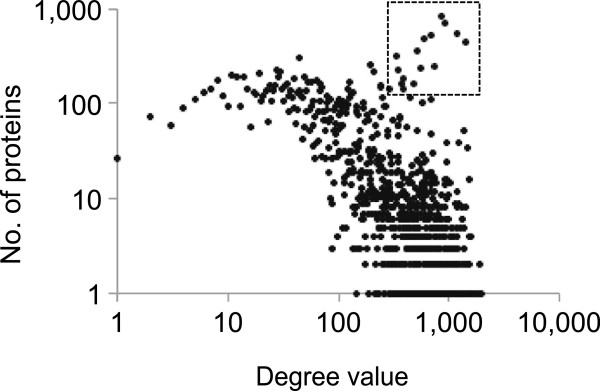
**Distribution of degree values in human PON.** The points in the box represent huge clusters of proteins with the same domain composition.

For each PON, we investigated three network properties for their largest/main sub-graphs (Table 1), namely, average shortest path length, clustering coefficient, and degree. The average length of shortest path between any two nodes in a PON is in the range of 4 ~ 6 for the four species and decreases with the increasing network size (Table 1). This is consistent with other reports that the recombination of existing domains, rather than acquiring new domains, could be the majority mechanism to form new proteins and increase genome complexity[43,44]. Another key feature of the networks is their high clustering coefficients (>0.9), which indicates that the PON is a hierarchical network (Figure 2) composed of densely connected modules accompanied by a few long distance connections. The pattern of degree distribution of yeast, fly, worm, and human was found to be similar to each other despite the different network size. The number of nodes with a specific degree value did not follow a typical power law distribution of a scale-free network (Figure 3) and the logarithmic relationship between the two variables is not as linear as that for a domain co-ocurrence network [32]. There are huge clusters of proteins with the same high degree value in a PON (see examples in the upper right corner of Figure 3) and proteins in the cluster are often connected to each other with the same domain composition. The degree value of a node is correlated with the popularity of its domain components other than the total number of domains it contains. For example, in the human PON, the triple functional domain protein [UniProt : O75962] has the highest degree value of 1,983 but only contains 5 annotated domains, Spectrin, RhoGEF, SH3_1, I-set, and Pkinase, which prevail in a large number of nodes ranging from 91 to 930. On the other hand, the average degree value (257) for proteins with the most divergent domain composition (8 domains) is lower than the average (312) for the total network.

**Table 1:** Network properties of the main sub-graphs of four investigated genomes

	**No. of proteins**	**No. of domains**	**No. of GO terms**	**Mean value**		
				**Degree**	**Path length**	**Clustering coefficient**
Yeast	762	237	146	38.4	5.65	0.94
Fly	6,099	915	385	101.9	4.52	0.93
Worm	7,742	745	343	121.3	5.02	0.95
Human	25,455	1,478	551	312.5	4.14	0.92

The mean value was calculated for the degree and clustering coefficient of all nodes and the shortest path length between any two nodes in the network.

For the entire network of the four species, 65 ~ 75% proteins were annotated with at least one GO term according to the InterPro database and the mean value of the annotations was very similar (2.4 ~ 2.6) despite of the different genome size. The nodes in a PON sub-graph frequently have a common GO term annotation. About 30% of the nodes share the most popular GO term, *protein binding function*, for the main sub-graphs of the fly, worm, and human networks. On the other hand, the most popular GO term for the main sub-graph of the yeast is *ATP binding function*, which is shared by 41% of the nodes. Nevertheless, 95% of the nodes of the third largest sub-graph of the yeast network, which contains 106 proteins and 36 domains, share the most popular GO term *Protein binding function*. Compared to the main sub-graphs, other sub-graphs usually are small, comprise only a few domains, and have a very popular GO term among the nodes.

### Protein function prediction with a single-genome PON

We predicted the function of a protein by statistically analyzing GO terms associated with domains in its neighboring nodes, i.e. the domain-based method. Since the annotated GO terms in InterPro database were exactly the same for identical domains among various proteins, the mean success rate was extremely high (>99%) if the GO terms associated with the common domains of the query protein and neighboring proteins were favorably considered. The success rate was not 100% only because in some cases the query protein contained a few domains observed in none of neighboring protein. In real predictions, we might not know the functions of the query domains, and so in our prediction, only the GO terms associated with the other “not shared” domains were statistically analyzed and those GO terms with a high occurrence frequency were compared with the combination of annotated GO terms from all domains of the query protein. The success rates predicted with the single-genome PON for yeast, fly, worm, and human were 30.2%, 34.2%, 32.6%, and 40.0% (Table 2), respectively, and were much better than the corresponding rates (4.9%, 6.7%, 6.5%, and 9.9%) obtained in the control group of random predictions.

**Table 2:** Results of protein function prediction with a single-genome PON

	**Success rate**^**a **^**(%)**	**Predictable**^**b **^**(%)**	**Coverage**^**c **^**(%)**	**Top 1**	**Top 3**
				**accuracy**^**d **^**(%)**	**accuracy**^**e **^**(%)**
Yeast	30.2	37.1	33.9	34.0	63.0
Fly	34.2	54.1	47.4	37.5	59.6
Worm	32.6	53.0	38.3	36.0	61.0
Human	40.0	68.3	55.8	43.3	70.4

^a^Percentage of correctly predicted GO terms averaged throughout the whole genome.

^b^A node was predictable if its neighboring nodes were annotated with at least one GO term.

^c^Percentage of predictable and annotated nodes.

^d^The GO term with the highest frequency of occurrence was evaluated.

^e^The best of the top 3 predictions was evaluated in cases that neighboring nodes were annotated with at least three GO terms.

**Figure 4:**
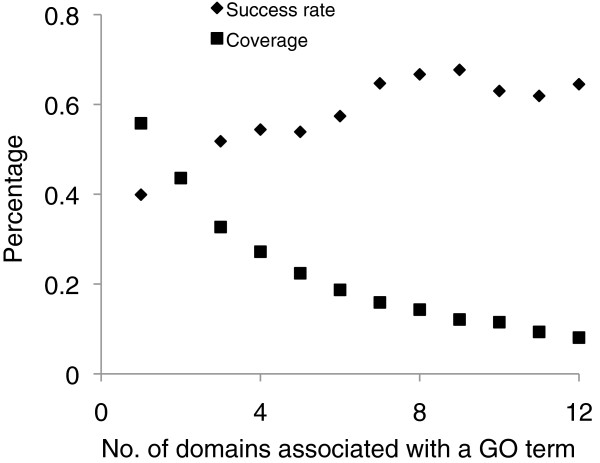
**Effect of domain diversity on function prediction accuracy.** A GO term was used for prediction only if it was associated with at least a certain number of domain types at the neighboring nodes.

One likely factor contributing to the high success rate for human as compared with yeast is the size difference between the two networks. For a query protein with domains **A and B** and its neighbors **AC1**, **AC2**, …, and **AC*n***, the more **C*n*** domains share a common GO term, the higher the possibility that domain **B** or protein **AB** will associate with the same GO term (Figure 4). In fact, the average success rate increased to over 60% and the best of top 3 accuracy was over 97.5% for the human PON if we considered only GO terms associated with at least 7 domain types (***n*** > =7). For the yeast PON, it is unlikely to identify GO terms associated with a large number of domains due to the small network size and the low degree value as seen from Table 1. Actually, the success rate of yeast (30.5%) was slightly higher than that of human (26.5%) for nodes where all analyzed GO terms were associated with one domain. The success rate slightly increased to 33.9% but the coverage significantly decreased to 16.1%, if we considered only GO terms shared by at least two domains in the yeast PON.

### Prediction with a composite PON

We constructed the composite PON by combining different genomes because the success rate of the domain-based method is positively correlated with the size of the network and the GO term annotation of a domain is exactly the same for various genomes according to InterPro database. The composite PON from the four species (yeast, fly, worm, and human) containing 74178 nodes and 5079 domains was constructed to investigate whether the prediction could be improved with a larger PON. The accuracies obtained for each of the four species using the composite PON were compared with those obtained using their individual single-genome PONs. For the three species with a smaller-sized genome, yeast, fly, and worm, the success rate was slightly improved by up to 4% and the coverage was also increased by 7%-12%. For the human genome, the coverage slightly increased by 1.2% though the success rate decreased by 1.6% (Table 2 & Table 3).

There are two advantages using the composite PON for protein function predictions: one is to improve the accuracy for those nodes that already can be predicted with the corresponding small-sized single-genome PON, and the other is to improve the coverage by adding new predictions, albeit at lower accuracy. With the composite PON, the success rate significantly increased by 8.7%, 2.6%, and 4.6% for those annotated and originally predicable nodes for yeast, fly, and worm, respectively. On the other hand, the new predictions that were unpredictable with a single-genome PON showed lower success rates (<20%) except for worm (32%), partly due to the lack of annotated domain types at the neighboring nodes. For example, the average occurrence frequencies of the most popular GO terms were 1.3 and 1.8 for the new predictions in the composite network compared with 6.3 and 6.2 for the originally predictable nodes for fly and worm, respectively. In future studies, more proteins and genomes may be used in the combination to improve the prediction accuracy and coverage even for the human genome.

### Prediction using the second layer neighbors

Frequently, only a few or no annotated domains were observed at direct neighboring nodes. To increase the coverage, we thus made predictions with the domain-based method considering the second layer nodes, i.e., the radius 2 neighbors of the query protein. For a query protein **AB** (**A and B** are domain or domain combination of the protein), the direct neighbor **BC**, and a second layer node **CD**, we made the prediction based on frequency counting of the GO terms associated with the domain **D** and all its peers. Here, for a clear comparison, we limited the domain **D** such that **D** is not a component domain for any immediate neighbors, i.e., **D** does not combine with **A** to form any protein. Usually, the accuracy is lower than that predicted from direct neighbors (Table 3 & Table 4). Nonetheless, if the composite network was used and only the GO terms associated with a lot of type **D** domains (more than 16, for example) were considered, an impressive success rate (>30%) and coverage (>30%) could also be obtained. For small genomes, it should be noted that the composite network is essential for the high performance due to the limited number of domains and domain connections in the single-genome networks.

**Table 3:** Results of protein function prediction with a composite PON of four genomes

	**Overall success rate (%)**	**Coverage (%)**	**Success rate (%)**	
			**High degree nodes**^**a**^	**Low degree nodes**^**b**^
Yeast	32.8	45.9	38.9	15.4
Fly	34.4	54.1	36.8	17.2
Worm	36.1	48.2	37.2	32.0
Human	38.4	57.0	38.8	19.4

^a^Annotated and predictable with a single-genome PON.

^b^New prediction with the composite PON.

**Table 4:** Results of protein function prediction with the second layer nodes

	**No. of domains associated with a statistically analyzed GO term**					
	**> = 1**		**> = 4**		**> = 16**	
	**Success rate (%)**	**Coverage (%)**	**Success rate (%)**	**Coverage (%)**	**Success rate (%)**	**Coverage (%)**
Yeast	20.6	41.5	26.1	30.2	28.2	17.6
Fly	24.7	51.7	28.4	42.8	33.2	30.0
Worm	23.5	46.6	28.1	37.1	34.7	24.9
Human	28.5	55.3	31.6	47.5	36.7	33.0

The composite network of four genomes was used for prediction.

### Prediction and evaluation with gene ontology annotation (UniProt-GOA) database

Proteins may acquire different functions in evolution while maintaining the same domain composition. There are inherent limitations of domain-based function prediction methods, where the function of a domain is fixed despite their existence in various proteins. With a PON, we are able to predict the function of a protein by statistically analyzing manually annotated GO terms for each neighboring protein. We obtained the GO term annotations from the gene ontology annotation (GOA) database for both prediction and evaluation. Using a single-genome PON, the mean success rate (60.8%) of yeast, fly, worm and human (Table 5), is much higher than that (48.9%) predicted by the domain-based method. Here, for fair comparison, GO terms associated with the common domains between the query protein and neighboring nodes were considered favorably with the domain-based method and the annotations from GOA database were also used for evaluation. Unfortunately, the success rate predicted with the GOA database decreased to 55.6% when the composite PON of four genomes was used for prediction and decreased further to 48.0% when the composite PON excluding the predicted genome was used (each query protein was treated as one node of the network while all other proteins belonging to the same genome were removed, e.g., the composite PON of fly, worm, and human was used to predict the function of a yeast protein). On the other hand, the mean success rate of the four species predicted by the domain-based method (46.1%) was nearly unchanged assuming no genome and domain annotation information to construct their own PONs.

**Table 5:** Results of prediction and evaluation with GOA database

	**Success rate (%)**	**Coverage (%)**	**Top 1 accuracy (%)**	**Top 3 accuracy (%)**
*Prediction with a single-genome network*				
Yeast	48.3	75.2	75.7	90.0
Fly	63.6	82.2	81.8	89.0
Worm	70.8	66.8	84.6	91.3
Human	60.7	84.6	80.6	90.8
*Prediction with the composite network constructed from the other three genomes excluding the predicted one*				
Yeast	35.5	85.0	68.0	86.1
Fly	51.1	84.8	77.0	85.8
Worm	56.0	67.8	77.5	86.2
Human	49.3	79.1	72.7	84.6

Moreover, the GOA database used for prediction is a comprehensive GO annotation dataset and the coverage is very high for the four species predicted with the composite PON of other genomes, respectively, achieving a mean value of 79.2%. As a comparison, the domain-based method yield a mean coverage of 71.0%, which is also high compared with those in Table 3 for considering the common domains in the prediction. Furthermore, we define the recall rate as the percentage of correctly predicted GO terms in total annotations of the query protein. The recall rate of the domain-based method (36.6%) is significantly lower than that of the GOA database method (47.5%) since the InterPro database used for the domain-based method is only one of information sources to build the GOA database and the predicted GO terms are often less than the annotations in GOA database.

In general, the GOA database method shows a better success rate, coverage of query proteins, and recall rate of observed GO terms than the domain-based method, especially in prediction with a single-genome PON. However, there are only 13 species with genome-wide annotations in the GOA database until the end of 2012, therefore the applicability is rather limited. It is also not clear to what extent the high success rate predicted with the GOA database is related to the sequence similarity between common domains of the query protein and neighbors. On the other hand, we optimized the domain-based method without considering the common domains. The unknown function of a domain can be predicted from other functionally characterized domains, which is especially helpful for biological experimentalists.

## Discussion

We built PONs for protein function prediction. Our function prediction algorithm is based on two observations. Firstly, if two domains (or domain combinations), **B** and **C**, combine with the domain **A** to form two proteins: **AB** and **AC**, it is possible that the domain **B** and **C** share the same GO terms. Actually, for all connected pairs, e.g., **AB** and **AC**, in the human PON, a GO term associated with domain **B** is also associated with domain **C** in 24.4% of cases compared with 4.7% in the random control procedure. In fact, this indirect functional association was also observed at protein level in early studies. Chua et al. found proteins interacting with the same partners had a greater likelihood of sharing similar physical or biochemical characteristics [14]. Secondly, a GO term that is associated with **A** is also the annotated GO term of **B** in 35.4% cases, i.e., domains within a protein may share the same GO term annotations, which is the basis for domain function prediction with a DCN [32]. It should be noted that the GO terms shared by **B** and **C** are not due only to the fact that **B** shares GO terms with **A** and **A** shares GO terms with **C** (35.4% × 35.4% <24.4%).

Wang et al. made protein function predictions by integrating the GO terms predicted for each of its domains using a single-genome DCN [32]. If all the conditions were equally set, the domain-based PON methods and the DCN methods should produce exactly the same results for protein function prediction. Nevertheless, PONs contain richer information than DCNs, which makes it possible to yield better prediction results. For example, the current Pfam 26.0 has a sequence coverage of only 69.7% and an amino acid coverage of 44.4% for the human genome [38]. For the DCN method, the prediction completely relies on the Pfam annotation. However, the complete GO term annotations of a protein could be obtained from GOA database [41] and used for function prediction with a PON even if only a part of the protein is annotated with Pfam domains.

GO term comparison is a yes-or-no binary criterion in this study even though two different GO terms may share functional similarity. Nevertheless, the mean value of success rates calculated with the binary criterion strongly correlates with that of the similarity scores in large-scale calculations like those presented here. For example, the mean success rates predicted and evaluated with the GOA database were 48.3, 41.1, and 35.5% respectively, for yeast proteins using the single-genome PON, the composite PON of four species, and the composite PON excluding yeast. When the semantic score between the predicted GO term set and the annotated set of each protein was calculated with the online method G-sesame [45], the mean values correspondingly increased to 0.709, 0.678, and 0.638, respectively.

The currently proposed methods can be used for the prediction of new functions of a protein in addition to the annotated GO terms, or a protein with unknown function. In fact, there are 3.2% yeast proteins that are not annotated but predictable with the domain based method using the single-genome PON (Table 2). The number increased to 7.5% by using the composite network of yeast, fly, worm, and human. In future, more genomes could be used to build the composite network to improve the prediction accuracy and coverage for the domain-based prediction method. Results from multiple approaches such as the domain-based method, prediction with the GOA database, and analysis of the second layer neighbors could also be combined to produce a comprehensive prediction.

## Conclusion

The protein overlap networks have different properties compared with the domain co-occurrence networks. The PON of an entire genome contains many disconnected sub-graphs with one main sub-graph, which comprises 50% of proteins except for the relatively small PON of yeast. The logarithmic relationship between the number of nodes and the corresponding degree value does not follow a typical power law distribution of scale-free network. The clustering coefficient of the networks is exceptionally high (>0.9). Using a PON, protein functions can be predicted with the domain-based method or gene ontology annotation database. The GOA database method usually shows a better performance than the domain-based method. The composite PON of multiple genomes can be used to enhance the performance for the domain-based method, especially in prediction for small genomes. But the GOA database method achieves the best accuracy for a protein when the single-genome PON of the same species is used. The success rate of the domain-based method is close to that of the GOA method in prediction for proteins without genome-wide sequence information and domain annotations. Moreover, the domain-based method can be optimized to predict the functions for an uncharacterized domain from the annotated GO terms of other domains.

## Abbreviations

PON: Protein overlap network; GO: Gene ontology; PPI: Protein-protein interaction; GC: Genomic context; DCN: Domain co-occurrence networks; SVM: Support vector machine; GOA: Gene ontology Annotation.

## Competing interests

The authors declare that they do not have any competing interests.

## Authors’ contributions

SL designed the study, implemented the algorithm and drafted the manuscript. DZ, HG, DMS, and CZ helped prepare the data and draft the manuscript. All authors read and approved the final manuscript.
